# Cybersecurity in ICT Supply Chains: Key Challenges and a Relevant Architecture

**DOI:** 10.3390/s21186057

**Published:** 2021-09-09

**Authors:** Xavi Masip-Bruin, Eva Marín-Tordera, José Ruiz, Admela Jukan, Panagiotis Trakadas, Ales Cernivec, Antonio Lioy, Diego López, Henrique Santos, Antonis Gonos, Ana Silva, José Soriano, Grigorios Kalogiannis

**Affiliations:** 1 CRAAX Lab, Universitat Politècnica de Catalunya, 08800 Vilanova i la Geltrú, Spain; eva@ac.upc.edu; 2 ATOS Research and Innovation, 28037 Madrid, Spain; josefrancisco.ruiz@atos.net; 3 Communications Network Group, Technische Universität Braunschweig, 38106 Braunschweig, Germany; a.jukan@tu-bs.de; 4 Sinelixis S.A., 14343 Athens, Greece; ptrak@synelixis.com; 5 XLAB d.o.o., SI-1000 Ljubljana, Slovenia; ales.cernivec@xlab.si; 6 Politecnico di Torino, 10129 Torino, Italy; lioy@polito.it; 7 Telefónica Investigación y Desarrollo, 28050 Madrid, Spain; diego.r.lopez@telefonica.com; 8 Centro ALGORITMI, Universidade do Minho, 4800-058 Guimaraes, Portugal; hsantos@dsi.uminho.pt; 9 Optimum S.A. Information Technology, 17674 Kallithea, Greece; agonos@optimum.gr; 10 Sonae MC Serviços Partilhados S.A., 4470-177 Maia, Portugal; amsilva@sonaemc.com; 11 Capgemini Engineering, 08005 Barcelona, Spain; jose.soriano@altran.com; 12 Sphynx Technology Solutions AG, 6300 Zug, Switzerland; g.kalogiannis@sphynx.ch

**Keywords:** cybersecurity, supply chains, IoT systems, systems integration, real scenarios analysis

## Abstract

The specific demands of supply chains built upon large and complex IoT systems, make it a must to design a coordinated framework for cyber resilience provisioning, intended to guarantee trusted supply chains of ICT systems, built upon distributed, dynamic, potentially insecure, and heterogeneous ICT infrastructures. As such, the solution proposed in this paper is envisioned to deal with the whole supply chain system components, from the IoT ecosystem to the infrastructure connecting them, addressing security and privacy functionalities related to risks and vulnerabilities management, accountability, and mitigation strategies, as well as security metrics and evidence-based security assurance. In this paper, we present FISHY as a preliminary architecture that is designed to orchestrate existing and beyond state-of-the-art security appliances in composed ICT scenarios. To this end, the FISHY architecture leverages the capabilities of programmable networks and IT infrastructure through seamless orchestration and instantiation of novel security services, both in real-time and proactively. The paper also includes a thorough business analysis to go far beyond the technical benefits of a potential FISHY adoption, as well as three real-world use cases highlighting the envisioned benefits of a potential FISHY adoption.

## 1. Introducing the Scenario

The unstoppable evolution of ICT systems, with innovative technologies and business models, is driving a massive digital transformation, turning into the Industry 4.0 revolution. At the same time, the larger a society’s dependence on ICT systems, the more critical the effects of any ICT infrastructure disruption will be. Today, the resilience of ICT systems is premium, and every ICT system is expected to implement at least a set of basic mechanisms to prevent, resist, and recover from any type of disruption in a timely manner, thus minimizing the impact on service quality and user experience. Particularly in complex ICT supply chain scenarios, the ICT implementation of physical supply chains, serving multiple actors in finance, manufacturing, healthcare, and many other sectors, not only individual parts of the supply chain need to be secured and reliably provisioned, but also the end-to-end process of securing the ICT supply chain. In real words, however, and according to an IBM/Ponemon study, 77% of organizations that individually or jointly participate in a supply chain process do not even have an incident response plan. The National Cybersecurity Alliance found that 60% of SMEs would be out of business within six months of being hit by a cyber attack [1]. On the other hand, the concept of cyber resilience is expected to become the norm, and one of the key measures of an ICT system’s ability to continue its operations in the event of a cyber attack (be it either software or hardware) or incident. According to the US National Institute for Standards and Technology (NIST), cyber resilience is defined as “*the ability to anticipate, withstand, recover from, and adapt to adverse conditions, stresses, attacks, or compromises on systems that include cyber resources*” [2]. Therefore, it is imperative today in achieving cyber resilience of any ICT system, which is particularly a challenge in the presence of disruptions, whether they be due to malicious security attacks or due to unreliable hardware and software system components and their implementations. 

### 1.1. Background

In practice, an efficient resilience strategy would ideally leverage the following three main components: (i) continuous availability, enabled by both the deployment of strategies to guarantee an “always-on” customer experience, and the required protection in front of disruptions; (ii) IT workload mobility, permitted by the deployment of strategies facilitating traffic offloading and resource migration in a distributed computing environment, including edge and cloud computing; and (iii) multi-cloud agility (also including hybrid clouds and coordinated edge-cloud), to determine the optimal set of resources that best match the expected level of resilience for each application. These three components are driving the deployment of the corresponding policies to provide security, trust, and performance guarantees, coupled with efficient network and compute infrastructure management strategies, in order to optimize the resource allocation, self-healing, and dynamic reconfiguration of ICT resources. Moreover, it is not the integration of these policies and strategies that must be pursued, but their effective coordination in complex multistakeholder ICT infrastructure scenarios. To this end, coordinated methods are also needed to forecast and accurately estimate vulnerabilities and risks potentially impacting the performance. In turn, these forecasts can be used to assess potential risk for security and privacy, and the related accountability and mitigation strategies, as well as the solutions to operate resilient services on top of potentially unreliable infrastructure components. It is, in fact, a major open challenge to providing these properties end-to-end, i.e., across the entire ICT supply chain.

A further challenge to be addressed in the context of cyber resilience is its strong link to cybersecurity. Cybersecurity is one of the greatest challenges of our era. In May 2017, the WannaCry malware cyber attack infected more than 200,000 computers across 150 countries, with the total damages estimated to be hundreds of millions of Euros [3]. During the same year, more than 26% of US healthcare consumers experienced a breach of their healthcare data, which included their social security number, contact information, electronic medical record, or health insurance ID [4]. This is hugely reflected and significantly amplified in the supply chain realm, among several other factors, because of the potential of the so-called domino effect. According to [5], there were reports of a worm “Stuxnet” that reportedly infiltrated Siemens industrial control software and later impacted the operation of an Iranian nuclear plant through the ICT supply chain. Also, in [6], it was reported that components of the Boing airliner were failing due to glitches in the Japanese supply chain production that globally affected airports and grounded airliners in India, Chile, and the United States. In [7], the authors reported, from their work in an EU project, that the major crimes encountered by supply chain stakeholders in Europe were theft in transit (23%), data theft/cybercrime (11%), bogus companies (10%), and insider fraud (10%). Also, other crimes were reported, including smuggling (9%), counterfeiting (9%), and terrorism (6%). Less frequent in the past, but possibly a bigger threat in the future, were also environmental crimes in the supply chain [8].

Finally, according to Appknox [9], the number of attacks in 2019 grew notably when compared to previous years. Moreover, even worse, the same study reports that “…*by the year 2020, the costs related to damage caused by cybersecurity breaches may reach $5 trillion and that is why it becomes essential to ensure that your business’ infrastructure is up-to-date and ready to ward off cybercrimes*”.

**Figure 1:**
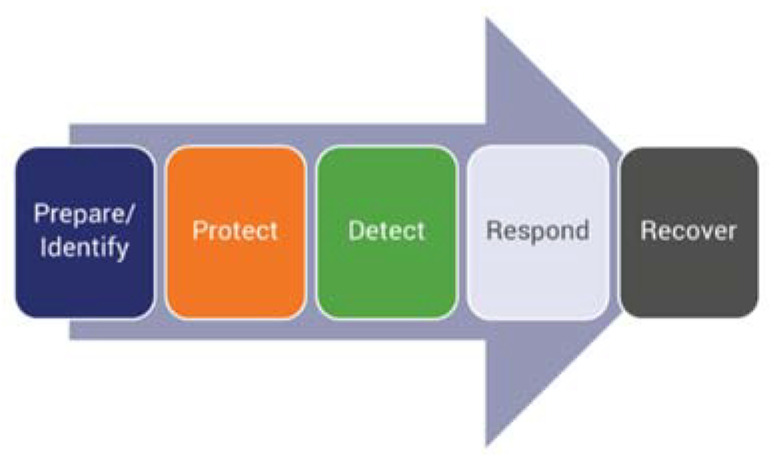
Five pillars for security evaluation.

These examples illustrate that cyber attacks affect the whole spectrum of services and the application domains simultaneously, and do not distinguish between e-health and social media services anymore, or industrial IoT and telecom operator networking devices, when choosing their targets. This alone establishes a strong interlink between cyber resilience and cybersecurity. Cyber resilience, while relying on cybersecurity, also assumes that not all parts of the system can be cybersecure, not only for economic reasons, but also for reasons of usability and scalability. Hence, establishing a proper link between cyber resilience and the fundamental pillars of cybersecurity is another challenge. To this end, a proper evaluation of the cybersecurity process is needed. For instance, Symantec [10] proposes a five-pillar approach to building the cybersecurity part of a cyber resilience plan (Figure 1).

Today, governments, enterprises, and individuals are systematically and collectively outpaced in managing their cybersecurity systems and appliances, by external attackers, internal threats, and fundamentally unreliable and unsecured components of ICT systems. The threat is not just basic sensitive information being stolen anymore, or a website being deactivated, but a plethora of quiet and unforeseeable threats, where attackers creep in and can change a system’s behaviour and network configuration at their will [11]. These attacks are polymorphic in nature and sophisticated, using previously unseen custom codes that are able to communicate with external command and control entities to update their functionality, or even implement themselves entirely from code fragments that they intelligently harvest from benign programs, scripts, and software blocks that are already present in the cybersecurity system in place [12].

### 1.2. Challenges

Due to the dynamicity and sophistication of the current cybersecurity and privacy threats, security administrators and IT operators must face the following five unprecedented challenges when trying to make their system resilient.

*Challenge 1: Need for end-to-end solutions for vulnerabilities and risks management*

With around 350,000 new malware programs appearing every day [13], and 88% of respondents [14] from medium-size companies replying that they receive up to 500 severe/critical alerts per day, it is evident that only a limited percentage (~1%) of the critical threats are analyzed, which indicates the lack of effective methods to characterize, detect, classify, forecast, and estimate threats, risks, vulnerabilities, and suspicious activities. On one hand, the challenge is not just detecting the vulnerability itself, but rather building an innovative solution to manage the whole vulnerabilities lifecycle, including the characteristics mentioned above, and the vulnerabilities propagation within the entire end-to-end supply chain too. On the other hand, cybersecurity and privacy risks must also be accurately estimated within the entire supply chain, to meet performance expectations through an appropriate data sharing mechanism [15], as well as to enable dynamic updates through real-time awareness of ICT systems’ actual states.

*Challenge 2: Lack of evidence-based metrics for security assurance and trust guarantees*

Security and trust assurance should not only be inferred from an observed absence of security incidents; this observation may be an indication of the absence of attacks during the monitored period or the incapacity of the system to detect attacks. Similarly, security assurance must not only leverage on trust, but on evidences supporting specific security claims. However, there is a lack of effective metrics to characterize composed ICT systems performance with regards to cybersecurity and privacy, thus making it difficult to provide the certification of complex systems. Similarly to how physical goods require metrics to distinguish between high-quality goods and low-quality goods (e.g., cotton, wood, or a car), the ICT supply chain also requires effective metrics, or else one cannot distinguish between highly secure and low-security parts in the supply chain. Consequently, it is necessary to define both the security claims and the set of metrics used to characterize these claims, and choose the proper evidence for each specific claim. In the context of certification, the former can be aligned with the objectives within the considered scope and target of the certification, and the latter can provide the necessary certification auditable evidence needed to verify the (continuous, where needed) compliance with the desired objectives. The mentioned evidence and metrics must be identified in the early stages of the ICT system design, towards a security and trust by design approach that optimizes the obtained results and minimizes the costs, while also considering the relevant certification and regulatory requirements of each of the covered domains, to ensure that the intricacies (both in terms of objectives as well as associated controls and needed evidence) are comprehensively covered.

*Challenge 3: Cumbersome coordination in multi-actor and multi-vendor supply chains of ICT systems*

Security analytics, and operation technologies and mechanisms are characterized by complexity, as organizations are dealing with 10 to 25 different technologies [16], and involve IT administrators, network architects, software developers, and several other roles within different contexts. Their interaction is complex and encompasses organizational structures as well as supply ICT chain processes to design, construct, manage, and protect the ICT supply chain. Given such a heterogeneous and complex cybersecurity ecosystem, the process of coordination and orchestrated management of security appliances to provide trusted supply chains, as well as acquisition and configuration of new tools by the network administrators and IT operators, becomes a challenging endeavor. One of the daunting demanding challenges is reflected in security policies that are often specified by people who are different from the software developers actually implementing them, which leads to misconfigurations and improper responses to threats and attacks, mainly due to the diversity of development contexts, multi-vendor security controls, and technology maturity levels. Gartner estimates that 70% to 99% of data breaches result not from external, concerted attacks, but from misconfigurations (human error, software update, or technical malfunction) of the affected IT systems [17]. Also, in an entire supply chain scenario, there is not a single user controlling the whole system, thus lacking strict identity management and accountability mechanisms.

*Challenge 4: Static cybersecurity networked configurations and dynamic systems audit*

Even when a security policy is successfully developed and implemented, the security systems in use are rather static with respect to the highly dynamic threat prevention and mitigation techniques needed. In most of the cases, *neither the network elements nor the security appliances support a reconfiguration framework to meet the pace of the highly dynamically changing nature of cyber threats*. The ease of attacking an ICT supply chain is largely due to the network. In fact, the network actually significantly amplifies the security threat in the supply chain. While newly proposed concepts of network function virtualization (NFV) and, previously, of software-defined networking (SDN) have opened up the window of opportunity to dynamically control the network, this has neither be used in connection with dynamic security policy enforcement nor for the effective protection against cyber threats; instead, it is quite the contrary [18]. The network challenge is further exacerbated when considering the influx of the resource-constrained nature of IoT devices and the myriad of potentially insecure devices to all be interconnected in a typically complex ICT system today. This requires innovative methods to developing resilient systems on top of a myriad of fundamentally insecure components and novel tools to better audit the different systems and interconnected components.

*Challenge 5: Unlikely wide adoption of integrated cybersecurity solutions for composed ICT systems*

While several commercial cybersecurity solutions exist, offering integrated solutions to network and IT administrators, integrated solutions, in general, only remain beneficial for specialized environments (military, governments, large financial player). In the commercial sector, integrated approaches are proven to be complex, and require large operational expenses, along with a high learning curve for human administrators of complex integrated systems. Indeed, training security administrators on how to orchestrate and implement security services on new systems, in a highly specialized platform solution, takes significant time and financial effort. In fact, these solutions are not designed to meet the requirements of fast-evolving, large-scale heterogeneous systems, as in typical supply chains built on top of complex ICT systems, or to facilitate the proper data sharing and performance metrics benchmarking for ICT systems among different stakeholders. Thus, new approaches are needed to facilitate a coordinated, rather than integrated, deployment of cybersecurity solutions, considering the complexity of supply chains putting together composed ICT systems from different stakeholders, handled by human resources with different levels of skills in ICT management.

### 1.3. Contributions

Taking into consideration the challenges identified above, in this paper, an architecture for cybersecurity provisioning in supply chains built on complex ICT systems is proposed. More specifically, the contributions of this work are as follows:

- A thorough literature review on the main relevant areas is given, including information security assessment, policy-based systems, trust monitoring, authentication, and threat anomaly detection, among others.
- The presentation of an innovative architecture, referred to as FISHY, designed to provide cybersecurity guarantees in the supply chains of complex ICT systems, consisting of the following: (i) innovative integrated services that are capable of managing the intent-based orchestration of security appliances through a well-defined toolset; (ii) an evidence-based security assurance and certification methodology; and (iii) a multi-party supply chain verification and forecasting system based on distributed ledger technology (DLT).
- A discussion on several business aspects, aiming at emphasizing the impact of the proposed architecture, as well as its potential adoption by key stakeholders.
- The introduction of three real industrial pilots, highlighting the theoretical benefits brought in by a possible deployment of the proposed FISHY architecture.

### 1.4. Outline

This paper is organized as follows: Section 2 revisits the literature in the closely related research areas that this paper focuses on. Then, Section 3 introduces the proposed FISHY architecture, along with its functional blocks. Section 4 describes the business scenario, emphasizing the involved stakeholders and potential market opportunities, as well as the factors that may hinder a wide adoption of the proposed solution. Next, Section 5 presents three real industrial use cases as potential supply chain scenarios benefitting from a FISHY deployment. Finally, Section 6 concludes the paper.

## 2. Review of the State-of-the-Art

This section revisits the current literature in fields closely linked to the proposed FISHY architecture.

### 2.1. Information Security Assessment

Information security assessment (or cybersecurity assessment) can be defined in different ways, according to the standards already available (mainly from ISO/IEC, CEN, and NIST). Some of them are focused on the devices’ security requirements accomplishment, others on the environment’s threat levels, and others on the effectiveness of the security control in place [19]. Those standards also help to characterize the assessment process, usually based on the technical analysis of the components (including vulnerability analysis), working tests (typically taking the component as a block-box), or just surveying functioning perception by operators [20]. Whichever method is used, a key central issue is always the quality of the metrics used (frequently constrained by observability). In fact, the security metrics problem has been researched in the last years and, despite some solutions for particular cases (such as smart grids or nuclear plants), there are no recognized generic models satisfying most implementations, particularly those where system diversity is the main characteristic, such as in the IoT paradigm [21,22,23].

A good metric should have some fundamental properties (i.e., objective, measurable, attainable, repeatable, accurate, and time-dependent), and it can be linked to several system dimensions, such as networks, software, users, and policies, eventually with a more fine-grained sub-classification scheme [22,23,24]. Several scientific contributions addressing the metric definition problem may be found in the literature, from ontological classification schemes to models supporting the metrics definition, such as the MDGSM (method for designing good security metrics) [22]. The subject was also targeted by well-recognized standards (e.g., ISO 27004 and NIST SP 800-53), which normally include application guides [25,26]. Finally, there are some attempts to use more complex multi-criteria solutions that explore relations and dependencies between different metrics, aiming to improve the decision-making process [27]. However, none of the aforementioned works can support an efficient set of metrics addressing the complexity and diversity present in IoT-based solutions.

### 2.2. Policy-Based Systems

Networks are traditionally configured (and reconfigured) manually, or with a very limited support from automatic tools. The rapid adoption of new IoT technologies has furthermore increased the ever-growing complexity and heterogeneity of modern IT infrastructures. Having a fully protected and efficient network in this scenario is thus becoming increasingly difficult, requiring the use of automatic tools to handle it in a timely and error-free manner.

To ease the pain of configuring a network, the introduction of systems that can automatically refine high-level security policies into either specific configurations or lower-level policies, has been already proposed in the current scientific literature. Very few papers exist on this subject [28,29,30], and the adoption of an automatic refinement workflow in production systems has been scarce to non-existent for several reasons. First, automatically translating high-level policies to lower-level policies or configurations is pretty difficult and requires a significant level of intelligence, unless the policies are very simple, or the landscape has a trivial architecture. Intrusion prevention systems (IPS), such as Snort [31] and Suricata [32], can be thought as a form of simplified policy refinement system, since they can be effectively configured to automatically use different reaction policies when an attack is detected. Despite the adoption of IPS solutions in production environments, their “refinement engine” only limits their usability in situations when the countermeasure is nearly trivial (e.g., drop all the suspected attacker packets). Second, translating a policy is not enough in complex scenarios. Once a set of security configurations is generated, it is also important for this set to be deployed in the right order, to prevent a temporary insecure state where the network security level may be too low. Virtually, no policy refinement system, as of today, offers this capability.

### 2.3. Trust Monitoring

Traditional strong integrity verifications of IT infrastructure nodes are performed on physical nodes via the remote attestation procedure. This procedure was standardized by the Trusted Computing Group [33], as a method to provide hardware-based integrity verification of an IT system, via an ad hoc chip named the TPM (trusted platform module). This strategy allows the continuous checking of the status of the software, services, and configurations deployed on a host [34,35]. This approach is, however, not necessarily ideal in highly virtualized environments, where most of the jobs are running into virtual machines and, especially, containers (lightweight virtual machines). In using this approach, in fact, virtual machines can be attested at deployment time, but cannot at runtime though.

While remote attestation allows the verification of the integrity of the software only, it cannot be used to check the traffic forwarded through the network. The classic way to detect unauthorized changes to the traffic flows is to make use of secure channels via specific protocols, such as TLS [36] or IPsec [37]. Although all these technologies ensure the confidentiality of a transmission (via encryption) or its authenticity/integrity (via digital signatures or MACs), unfortunately they do not verify if a packet was effectively sent, received, or traversed all nodes it was supposed to go through.

### 2.4. Authentication and Authorization/Security Requirement Management

It is widely accepted that the characteristics inherent to devices located at the edge of the network (such as the IoT devices) are making it difficult to provide security guarantees to their users, thus potentially hindering a large adoption of such devices to support innovative services. Although some contributions addressing this problem may be found in the literature, such as, for example, solutions based on the physical unclonable functions (PUF) concept [38], additional research efforts are still needed to suitably handle aspects such as the device mobility, heterogeneity, and low computing capacity, which may add serious risks to all scenarios where these devices are to be deployed. Thus, any system, platform, or solution leveraging IoT devices to run services must support several security requirements as those listed below [39]:

- Authentication: Edge devices must be authenticated to both the cloud (upper layer) and other edge devices (lower layer), allowing only authorized nodes to communicate and retrieve data. One of the main challenges here is to authenticate constrained IoT devices.
- Secure data sharing and data aggregation: Data sharing between the edge and cloud must be encrypted, and data aggregation in intermediate layers must be similarly managed. However, handling data sharing and aggregation in a distributed way demands for a novel security management approach to be designed.
- Secure service discovery: In order to only provide services to authorized users, services must be discovered and delivered in a secure manner, to avoid fake users and fake nodes.
- Malicious nodes detection: Distributed nodes are vulnerable to external and internal hardware or software attacks. Hence, a mechanism is needed to detect malicious nodes.
- Secure virtualization: Nodes must provide a secure virtualization environment to avoid malicious virtual machines, virtualization attacks, as well as to prevent an attacker to take control over either the hardware or the operating system, to launch attacks.

All these requirements must be met in a highly heterogeneous environment, where multiple nodes (IoT devices) are unstoppably joining and leaving.

### 2.5. Threat and Anomaly Detection

The automatic detection of traffic anomalies and network cyber attacks is not a novelty. Intrusion detection systems (IDS), such as Snort, Bro [40], and Suricata, are frequently used in production IT infrastructures. They usually detect threats by looking at specific patterns in the traffic, using advanced pattern matching rules. IDS are not trained, but are configured by experts with ad hoc pattern matching expressions, thus limiting their effective usage for at least two reasons. On one hand, writing detection rules for new attacks requires a significant amount of expertise and knowledge about a threat. On the other hand, zero-day attacks and recently discovered ones can pass through an IDS undetected, unless their fingerprint is very similar to another one in the intrusion detection system internal database.

To overcome such limitations, the current scientific literature started using supervised and unsupervised machine learning approaches to provide trainable attack detection tools with high accuracy. However, the current state-of-the-art is mostly focused on detecting anomalous traffic [41], without classifying the attacks, and the few articles devoted to attack classification are mostly limited to denial-of-services and volumetric attacks [42], as well as hazard detection and differentiation [43].

### 2.6. Threat Intelligence and Information Sharing

Security information and event management (SIEM) solutions aim at providing real time analysis and management of security alerts. They are commonly used in production environments, to have a global picture of the security status of an IT infrastructure, and can allow administrators to perceive a threat before it can maximize its damage [44].

Despite that Internet of Things devices are starting to become ubiquitous, unfortunately, traditional SIEM systems have limited capacities to interface with IoT devices and embedded systems. Consequently, research efforts are required to facilitate SIEM operations in IoT-based scenarios. One potential improvement may reside on minimizing the number of possible false positives, through improving SIEM import capabilities by facilitating SIEM to receive relevant structured data from multiple data sources. To this end, MISP (malware information sharing platform), along with the addition of the trust and reputation module, which will perform the needed analysis and enrichment before injecting the data into the SIEM itself, may be adopted. Another area of improvement would refer to the possibility of extracting new IDS rules from these enriched events through MISP, later to be dynamically sent to the SIEM, thus exploiting the built-in sharing capabilities of the former.

### 2.7. Identity Management and Accountability

The current identity management (IdM) systems are mostly based on centralized solutions, such as corporate directory services, domain name registries, federated services, or certificate authorities. However, these approaches are facing several issues, being fragmented and siloed between various service providers, thus limiting the adoption of a holistic view and delivering poor user experience. The upcoming reliance on billions of IoT devices makes it untenable to have all those devices controlled by a centralized identity provider, since a breach of this provider would be disastrous not only for revealing personal data and misallocation of virtual resources, but also for attacking the physical infrastructure, including the IoT devices.

The emergence of distributed ledger technology (DLT) offers a promising solution, easing the deployment of fully decentralized identity management strategies [45]. This technology pushes the ownership of identity away from centralized services to the edges, i.e., to individuals, so that the identities themselves are in control [46]. In this way, distributed ledgers provide a mechanism for managing a root of trust, with no need for a centralized authority, thus removing the single point of failure issue. Recently, DLT-based IdM solutions have been classified into the following two main categories: self-sovereign digital identities and decentralized trusted identity. The solutions in the first category offer self-sovereign identity through block-chain technology, where the owner has control over what information they share, without any external administrative authority [47]. Differently, the second set of applications offers a centralized service that provides identity proofing through existing identifications, such as a passport and driving license. With respect to the self-sovereign approaches, there are already a few of them providing authentication and authorization capabilities. Bitid [48] is an open protocol that allows simple and secure user login to cloud/web services, by authenticating the user based on the public key and block-chain-based network. The authentication proves the identity of the user to a service by signing a challenge. OpenID [49] is an open protocol that allows a user to authenticate to multiple services without the need for creating multiple different identities and passwords. It provides one unique identity to the user from some trusted identity provider, which can be used to sign into other OpenID-enabled services. Based on OpenID, NameID [50] is an experimental technology, which allows a user to register names that can be associated with the user data. These data can be verified by everyone in the block-chain network, but cannot be forged or censored by unauthorized attackers, and no one can retrieve the data without explicit user consent. Finally, uPort [51] is a platform that allows end users to establish a digital identity, which can be used as a user identity across multiple services, without any password. It gives full control of the user’s sensitive data to the user, by allowing users to own and control their digital assets, as well as to securely and selectively disclose their data to counterparts to access digital services. Moreover, it allows users to digitally sign and encrypt documents, data, messages, transactions, and to send these contents over the distributed ledger network to interact with decentralized applications.

### 2.8. Intent-Based Services

The automatic network management can reduce the network administrator’s tasks (network configuration, configuration change, etc.), and may leverage the concepts of policy or intent.

Policy-based network management (PBNM) [52] is a technique that enables the updating of network configurations with network administrator’s policies. PBNM enables policies to be defined, which manages network resources and ensures that network resources are appropriately allocated to users. Policies are formulated using the event–condition–action (ECA) rule and are described using the “if condition then action” rule. The common open policy service (COPS) [53] protocol has been standardized in the Internet Engineering Task Force (IETF). It has a simple query and response form, and it exchanges policy information between a policy server and its clients. Recently, the Simplified Use of Policy Abstraction (SUPA) working group has discussed data models of policies in the IETF. In the conventional management of network states, the simple network management protocol (SNMP) has been widely deployed based on a request–response form. Recently, the network configuration protocol (NETCONF) [54] has been discussed in the IETF NETCONF working group. The NETCONF is a management protocol for correcting the states of network devices and updating their configuration, and is based on an XML form. Yet another next generation (YANG) [55] is a data modelling language that is used to design configuration and state data on the NETCONF protocol.

The concept of intent-based networking (IBN) has been proposed as a new network management framework in OpenDaylight network intent composition [56]. An intent-based interface has been pursued rigorously by IETF, and major open-source project communities (ONF [57], ONOS [58], and OpenDaylight [59]) are working to provide a standardized intent-based northbound interface for SDN. An intent of a network administrator is used to be expressed in the concrete description of configurations stored on devices, to update configurations. To describe the intent, the concept of the intent-based network modelling language has been discussed in IETF IB-Nemo [60] BoF, and a draft specification and implementation of it is developed in the NEMO project [61,62,63]. Another specification method was also developed by policy graph (e.g., PGA [64]).

### 2.9. Artificial Intelligence

Network management and orchestration can require real-time (i.e., latency around milliseconds) complex decision making as softwarization and virtualization of network resources. Using artificial intelligence (AI) techniques enable historical, temporal, and frequency network data to be analyzed. Indeed, artificial intelligence techniques, especially machine learning (ML) and statistical learning algorithms [65], can help the FISHY framework to be intelligent as well as autonomous, i.e., to make network self-aware, self-configurable, self-optimization, self-healing, and self-protecting systems [66]. Simultaneously, the AI-enabled functionalities taking advantage of intent-based networking, NFV, SDN, network slicing, and security, will enable cognitive network management for 5G and beyond. The current development of network management solutions, including CogNet, Selfnet, SONATA, and 5GeX [67], are focused on cognitive network management for 5G devices. Thus, the work towards beyond 5G management solutions would require an optimizing network as an entity in a secure, resilient, and cognitive IoT-fog-cloud infrastructure, taking advantage of in-network computing and communication to minimize the overall energy footprint. However, the success of an intelligent and autonomous system is defined by the AI techniques that can effectively be adopted in different parts of the network management infrastructure. Thus, the intent orchestrator needs to provide not only the handcrafted policies, but should also utilize the power of big data and computing dynamic resources, making intelligent decision based on the processed data near the end users, providing low latency, as well security, as required by critical surveillance, medical applications, and many commercial applications [68]. Moreover, the work proposed in this paper, towards defining the FISHY architecture, will exploit natural language processing (NLP), i.e., the science of extracting the intention of text and relevant information from text, to support the management of intents by the intent-based resilience orchestrator block. Some popular “NLP as a service” platforms are as follows: (i) LUIS.ai [69] by Microsoft; (ii) Wit.ai [70] by Facebook; (iii) Api.ai [71] by Google; and (iv) Watson [72] by IBM.

For the sake of illustration, Table 1 summarizes the review of the art in the research fields related to the proposed cybersecurity solution.

## 3. Architecture for Cybersecurity Provisioning

### 3.1. Concept and Approach

The proposed FISHY architecture aims at delivering a coordinated cyber-resilient platform that would provide the appropriate set of tools and methods towards establishing trusted supply chains of ICT systems, through novel evidence-based security assurance methodologies and metrics, as well as innovative strategies for risk estimation and vulnerabilities forecasting leveraging state-of-the-art solutions, leading to resilient complex ICT systems, comprising the complete supply chain, particularly focusing on the IoT devices at the edge and the network systems connecting them.

Addressing the challenges 1 to 5, the proposed architecture is not envisioned as an incremental integrated cybersecurity solution, but rather as an extensible and programmable framework that can flexibly orchestrate the whole set of ICT systems and security controls. The aim is to provide an innovative cyber resilience framework, where complex ICT systems performance in an entire supply chain may be analyzed, in terms of the security, trust, and privacy impact on performance. To this end, the proposed architecture seamlessly combines advancements in several domains, including software-defined networking (SDN), network function virtualization (NFV), intent-based networking, AI-based techniques, and distributed ledger technologies (DLT).

**Figure 2:**
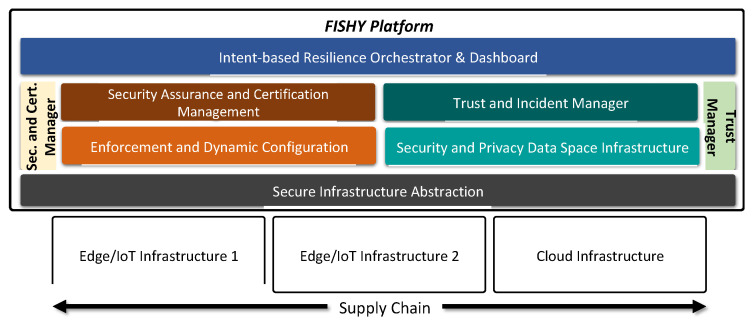
The technical overall concept.

The high-level architecture is depicted in Figure 2, where the entire supply chain, including the involved stakeholders, is also shown. Each stakeholder participates in the supply chain through resources and infrastructure, from data to IT infrastructure, either as provided by the stakeholder itself or reachable through other stakeholders via core network and clouds. The main concept relies on designing a security, trustworthy, and certification layer, transversal to the whole set of stakeholders in the supply chain, intended to make the entire ICT supply chain system resilient, but also to correctly measure the complete security compliance and consequently trigger the required actions (mitigation, reconfiguration, etc.), making sure that guarantees for a certain level of cyber resilience are provided. It is worth mentioning that the proposed solution is envisioned to be deployed on the entire set of devices and systems in the supply chain, most notably including the IoT ecosystem. The latter includes heterogeneous IoT devices at various localities and assumes their connections to gateways or hubs, edge, and cloud systems, as well as the network infrastructure to connect them all. Figure 2 also introduces the proposed functional architecture, where the following four principal functional modules are proposed: intent-based resilience orchestrator and dashboard (IRO), security and certification manager (SCM), trust manager (TM), and the secure infrastructure abstraction (SIA). The figure also shows the key blocks within the SCM module, namely, the secure assurance and certification management, and the enforcement and dynamic configuration, as well as the trust and incident manager, and the security and privacy data space infrastructure, both into the TM module. Starting from top to bottom, the intent-based resilience orchestrator and dashboard (IRO) module is designed to work as the user-centric interface, which is responsible for translating and orchestrating input actions into intents, to be used by other components. The security assurance and certification management is responsible for the provision of the auditable, evidence-based evaluation and certification of the assurance posture of complex ICT systems, based on identified security claims and metrics, setting the roots for the definition of a pan-European process for the certification of devices, processes, and systems, as required in today’s European market. The trust and incident manager provides tools for assessing the security of the stakeholder’s device, component or/and system. The enforcement and dynamic configuration block is responsible for making the entire system cyber-resilient, even when including potentially insecure components, based on the concepts of dynamic self-configuration. The security and privacy data space infrastructure is responsible for the collection and storage of data generated from the devices, processes, and components of the stakeholders’ ICT systems, being part of the supply chain. Finally, secure infrastructure abstraction (SIA) is the infrastructure-centric interface, and it works as a data interface between different edge/IoT or cloud infrastructures and the FISHY platform.

**Figure 3:**
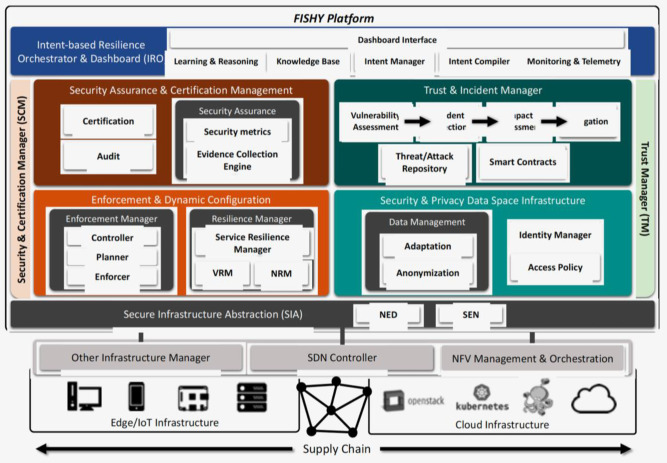
FISHY functional architecture in the entire ICT system.

A more detailed description of each individual module in the architecture is depicted in Figure 3, also including the interaction with the infrastructure along the whole supply chain. Indeed, the whole set of individual components within the modules and blocks defined in Figure 2 are represented in Figure 3. Each module, block, and component are described next, to facilitate the overall understanding.

### 3.2. Intent-Based Resilience Orchestrator & Dashboard (IRO)

The intent-based resilience orchestrator and dashboard (IRO) aims at automating the processing, storage, and management of intents, using natural language processing (NLP) into security workflows, which will be translated to security functions within the FISHY architecture. The processing and optimization of intents use AI, while keeping the human-in-the-loop, depending on the desired level of automation, in order to control and enforce a specific workflow that is able to react to new threats. The intent-based resilience orchestrator is divided into six main components, the dashboard interface, learning and reasoning, the knowledge base, the intent manager, intent compiler, and monitoring and telemetry. The main objective of the dashboard interface is to provide a unified, harmonized, and consistent application, interfacing the human serving as security administrator and the FISHY platform, showing as services, high-level policies, risks and vulnerabilities exposure, warnings, performance, metrics, etc. The inputs entered by the users of the dashboard will be managed by the rest of the components in the IRO. The learning and reasoning module receives rules or metrics from other blocks (e.g., TIM) and uses AI techniques to learn from the experience acquired in previous executions (e.g., considering how the ICT systems react to security alerts, which policies fit better to different scenarios, and learning from feedbacks from other modules), to predict the best decisions to be made, and to help the FISHY administrator understand which policies to choose. This component generates recommendations for the infrastructure operator, to drive automation to dynamically fix policies and optimize the performance of the intent manager. The knowledge base stores the relation between intents, corresponding workflows, and security policies. The intent manager is responsible for handling the intents, while checking the conflicting policies and guaranteeing the optimal implementation, depending on the dynamic rules chosen by the infrastructure operator. The intent compiler deploys the configuration obtained from the intent manager and will feed other modules in the FISHY architecture. Finally, unlike the current commercial solutions, our implementation of the monitoring and telemetry component is as follows: (i) able to dynamically monitor deployment changes enforced by continuous dynamic scheduling, provisioning, and auto-scaling; (ii) lightweight, yet effective and non-intrusive; and (iii) independent of any specific infrastructure technology. FISHY will containerize a monitoring and telemetry solution, collecting and storing data from different sources, including NFV infrastructure monitoring, Kubernetes infrastructure monitoring, VNF monitoring, SDN monitoring, etc.

### 3.3. Security Assurance and Certification Management

Security assurance and certification management (SACM) is responsible for providing an auditable, evidence-based evaluation and certification strategy for the assurance posture of complex ICT systems, based on identified security claims and metrics, also intended to boot strap the development of new models and tools that would lead to the definition and future establishment of a pan-European process, to be followed for the certification of devices, processes, and systems in the European market. The set of security metrics to be applied at the device, component, and system level are stored in the respective component, while the security assurance component is utilized for the proper configuration of the tests to be executed. The real-time, continuous assessment of the security posture of the complex ICT systems will be enabled by a purpose-built evidence collection engine, which will be responsible for aggregating the required evidence from multiple sources related to the operation of individual components, as well as the overarching processes that these components are involved in. This functional group of modules will also include audit and certification functions, leveraging the evidence-based approach of the assurance solution integrated into the FISHY platform. The certification block will provide evidence-based security, reporting, and certification to the needs of different stakeholders, ranging from senior management to external auditors and regulators, incorporating different access levels to the respective users. Finally, the audit block will be responsible for initiating, coordinating, and reporting to the IRO dashboard the auditing process results.

### 3.4. Enforcement and Dynamic Configuration

The enforcement and dynamic configuration (EDC) block is responsible for both making the supply chain measurably reliable end-to-end, and assessing the reliable and secure operation, even in the presence of potentially insecure components, based on the concepts of dynamic self-configuration. The general approach includes a predefined set of security features, based on an agnostic feature description language. This taxonomy allows the identification and translation of dynamically intent-based cybersecurity responses into specific configurations. Configurations are applied simultaneously at the network topology level and at each network security function (NSF) configuration leveraging the NFV technology. The main components in this functional block are the controller, planner, and enforcer. The controller is a network controller, mapping from the network-specific cyber threat solution to the actual NSF deployment and configuration. It can implement changes to the edge network topology and to the configuration of the running NSFs, based on the centralized FISHY intent-based resilience orchestration. This element will rely on an existing NFV orchestrator (NFVO) northbound interface, mapping the intent-based security policies to be translated and enforced on it. The register and planner is the component where the NSFs will register their security capabilities to be used in enforcement actions, using open standard interfaces, such as I2NSF [73]. The planner will use this information to combine and decide the best NSFs to use, their topologies, and the configurations to apply. Finally, the enforcer is the lower-level block of the EDC, continuously reconfiguring the whole ICT system via the existing NSFs, based on the available capabilities. This block will use standard (I2NSF) interfaces to NSFs whenever possible and support specific ones when no standard is available.

### 3.5. Trust and Incident Manager

The trust and incident manager provides the tools to be used for assessing the security of the stakeholder’s device, component or/and system. The vulnerability assessment tools will move beyond state-of-the-art (e.g., w3af [74]), providing, among others, automated vulnerability and risks analysis, or estimation and detection in source codes using deep representation learning techniques. Indeed, the functionalities of this module cover the following three important sub-processes: (i) determining and establishing assets on the infrastructure; (ii) determining, naming, and prioritizing the vulnerabilities found in the analyzed system, component, or environment; and (iii) proposing the most effective mitigation actions. The vulnerability assessment will be in charge of providing the insight of how the detected vulnerabilities may entail a risk, and understanding the degree of weakness that the monitored infrastructure may present. Applying this to the FISHY supply chain platform will make supply chains more resilient to threats and, more specifically, to vulnerabilities. Moreover, although several kinds of vulnerability assessments (performed on network, host, database, applications, etc.) may be found, from the FISHY perspective, an assessment of the monitored ICT platform for the entire supply chain would make more sense, given that supply chain platforms are usually made up of various components. Consequently, it would also be appropriate to assess IoT devices if they are going to contribute to the ICT infrastructure of the supply chain. Incident detection tools will be based on the outcome of the vulnerability assessment and will be based on machine learning techniques. This component will provide smart processing based on the collected data, thus covering several different research areas. FISHY plans to integrate incident detection into a holistic process of cybersecurity hardening, increasing resilience and enabling faster response time to incidents over the whole ICT infrastructure of a supply chain, by leveraging existing open-source technologies, such as Wazuh [75], and integrating and expanding the capabilities of the XL-SIEM (cross-layer security information and event management), an event management tool that is oriented around enhancing normal SIEM capabilities [76]. In FISHY, the functionality of the impact assessment block is oriented around defining and outlining the existent relation between the status of the system and the changes happening, involving the employment of both qualitative and quantitative data, which are normally expected to be faced to various indicators within the assessed item. Indeed, this block will help in determining how and to what extent the supply chain will be affected should a change happen in the overall platform. The functionality of performing the assessment within this block will be guided and assisted by cybersecurity tools, such as the risk assessment engine (RAE) [77], as they can enhance the results in terms of accuracy, saving time, and reliability. The mitigation component should be responsible for limiting the scope of the expected impact analyzed on the impact assessment component, by detecting anomalies from network/IoT data based on machine learning algorithms. In FISHY, the mitigation mechanisms based on ML algorithms are proposed to work in the following two different ways: online mode and offline mode. The threat/attack repository will store the outcome of the trust and incident manager module whenever the analysis leads to a threat or attack (be it software or hardware). The tools to be used to develop this block are still to be decided; it is recognized that some repositories already exist and that data sharing will be highly useful. Based on the immutability principle, the repository will store the result, so the information may be used for the expected evidence-based assessment, and also timely informing of other involved stakeholders. Finally, the smart contract is the realization of the component that would alert the stakeholders when a security-related service level agreement is violated.

### 3.6. Security and Privacy Data Space Infrastructure

The security and privacy data space infrastructure is responsible for the proper collection and storage of data generated from the devices, processes, and components of the stakeholders’ ICT systems, being part of the supply chain. It is based on the concept of the distributed and decentralized data storage concept (e.g., IPFS or data lakes), in which users hold a portion of the overall data, creating a resilient system for data storage and sharing. The data adaptation component is responsible for the homogenization of data coming at different intervals, in different data models (XML, JSON, small chunks of sensor data, logfiles, etc.) and following different communication means (REST APIs, Pub/Sub, etc.). Moreover, the identity manager is based on DLT, and is responsible for authenticating the users/processes connected to the secure and distributed data space, while the access policy component caters for preserving privacy per user accessing the data, according to specific policies set by the stakeholder responsible for the dataset. In this respect, not all users can access the whole set of data. Finally, the data anonymization component takes care of the privacy of the dataset shared by the stakeholders.

### 3.7. Secure Infrastructure Abstraction (SIA)

The main goal of the secure infrastructure abstraction (SIA) is two-fold. On one hand, it is intended to endow IoT systems with as many security guarantees as possible, assuming the inherent trend for IoT or edge devices to be potentially insecure. Two components are considered. The secure edge node (SEN) [78] is a software component designed to reside at the edge layer, and aimed at providing, by default authentication to IoT/edge devices, leveraging of an extensible blockchain architecture. This architecture provides a totally distributed and fault-tolerant chain of trust to IoT/edge devices, to be used to verify device signatures and establish secure TLS connections between the devices. The network edge device (NED) element will be in charge of controlling the network access of the protected environments, providing assurance for traffic flows, and ensuring a proper deployment and topology of the necessary monitoring and threat response functions. Security decisions and actions, as defined by any FISHY component, will be translated into an enforcement configuration in the NED, whenever appropriate. On the other hand, the secure infrastructure abstraction provides the proper means to the enforcement and dynamic configuration, and the trust and incident manager to interact with the NFVI resources, regardless of the particular technologies that are to be used (OpenStack, Kubernetes, AWS, OpenDaylight, ONOS), SDN controllers, or other infrastructure managers. A technology agnostic view of the infrastructure is foreseen in the proposed FISHY architecture. To this end, exposed API endpoints can be used for the management of the network services and VNF instances. The APIs can be further used to collect monitoring data from the NFVIs and the network services, providing useful information about the infrastructure status, allocation of resources for service deployment, VNF performance, etc.

## 4. Key Business Aspects

### 4.1. Market Considerations

Cybersecurity is an expanding business in recent years, mainly because IT infrastructure has been the focus part of cyber attacks, especially when more and more workloads are moving to cloud and edge, and the risk will continue to grow with the IoT adoption. Moreover, based on OVUM [79], less than 15% of organizations have developed a proactive approach to cybersecurity and digital risk, paving the way for innovative solutions, such as the one proposed in this paper.

Additionally, edge computing is ramping up in the technology market, with promising revenue streams [80], where operators have a key role, either as edge locations owners, as edge connectivity enablers, or as application enablers in edge. None of these potential strategies will succeed without considering resilience and security.

The unprecedented success of the internet as, not only a global communication tool, but also a powerful and indispensable mean to make business anywhere in the globe, has sparked the fast and unstoppable evolution of ICT networks, with 5G and IoT as the current main paradigms. Such a hectic pace has brought serious security issues. This, together with the increasing value of the digital assets involved in the daily business processes of companies and the clearer multistakeholder nature of the supply chains, makes ICT networks an appealing target for cyber criminals. Cyber attacks evolve quickly, and their potential effects are more and more dramatic, to the extent that they could damage entire countries, disrupt national economies and degrade standards of living that were taken for granted. In this eerie scenario, the need for cyber-resilient ICT networks pushes the market. New cybersecurity products must address the need to anticipate the threats, to absorb the impact of those threats, and to respond dynamically to ensure business continuity. The impact of IoT makes the number of devices connected to grow fast; therefore, the volume of data flowing skyrockets, and, in consequence, tracking suspicious data and anomalies becomes very difficult. These challenge the sensing capabilities of the organizations. Besides, privacy and cybersecurity are not a plus anymore, but a responsibility and duty for all the actors of the multistakeholder environment. According to a survey conducted by EY [81], business continuity and disaster recovery resilience have become a high priority for 57% of the respondents, data loss prevention for 56%, and security awareness and training as a means to gain cyber resilience for 55%. There are standards and approaches to cyber resilience promoted by industry, such as ISO 23316, which covers the principles and guidelines for organizational resilience. The public sector, and particularly national governments, are becoming increasingly involved. Such is the case in Scotland, for instance, whose government has created a 3-year program (2018–2021) with a set of specific actions pursuing to grow its national cybersecurity industry [82].

The proposed FISHY architecture is perfectly aligned to this context of strong market demands for products helping to increase cyber resilience, which is posed from both the private and public sector, as referred above. FISHY responds to all these needs, providing means to achieve higher cyber resilience guarantees. On top of this, it enables automatic responses, providing the needed intelligence to carry out the self-reconfiguration of the network without the intervention of operators, increasing the promptness, and thus diminishing human errors. Considering the multistakeholder aspect of supply chains, FISHY will also address the issues derived from the cascading propagation of the consequences of an attack, also focusing on mixing software and hardware attacks and the effects they both may have on the articular requirements of multistakeholder supply chains.

The different stakeholders will benefit from the genuine features of the FISHY components. Indeed, a successful FISFY platform adoption will open new business opportunities on added-value security services, which will become strongly demanded in the market. In short, (i) FISHY will bring several competitive advantages to network service providers (NSPs), allowing them to address the requirements of different customers and sectors, to expand their share in the value chain, evolving from network and connectivity providers to cybersecurity service providers; (ii) technology providers and ICT system integrators will benefit from FISHY, by means of new and more open ways of collaborating with their network provider customers, thus facilitating their strategic position and gaining an early-entrant advantage within the market of global cybersecurity solutions; (iii) vertical industries will become better enabled to apply advanced security mechanisms to their supply chains, with a better understanding of their features and how their particular requirements are addressed, as well as with the possibility of an open and independent verification of security policies, their enforcement, and their associated SLAs; and, finally, (iv) small and medium enterprises will acquire competitive advantages based on their business and market segments, and will be provided with a unique opportunity to extend their offerings and business advantages, towards an ICT global cybersecurity landscape, supporting new business models to push forward the project’s innovations to key security stakeholders.

### 4.2. Potential Stoppers

Although the benefits brought in by the proposed FISHY architecture, in terms of cyber resilience provisioning, are quite clear and relevant, some stoppers may hinder a wide adoption of the proposed solution. Particularly, factors that may influence the real FISHY relevance include the following:

- A low level of awareness about the need for clear responses to cyber threats, often due to a false sense of security, especially at the management level, including CEOs and boards of directors, those who have the power to decide on cybersecurity investments.
- Low levels of economic growth, insufficient funding, and unexpected interruptions for future and ongoing security innovation initiatives. In the long run, a lack of political support for these initiatives could be reflected in insufficient funding, but also in a failure of efforts devoted to deploying more-advanced security management capabilities.
- Misalignments in the technical and business evolution of some of the following potential core application environments: energy, industry, health, etc., also including network service provisioning itself.
- A changing regulatory landscape in network service provisioning and/or security, as a response to the development and progress of novel business models and business opportunities in the new networking market.

## 5. Proposed Use Cases

The FISHY architecture is designed to facilitate the deployment of a cyber-resilient platform for supply chains of composed ICT systems. To that end, several real industrial use cases are considered, with two main contributions. First, to assist on the definition of real industry requirements, in terms of trust and cybersecurity, thus guaranteeing the alignment of the FISHY design to the real needs. Second, to provide a real scenario where the FISHY architecture may be deployed, tested, and validated, also emphasizing the potential benefits of such a deployment. In this section, we present three potential use cases, i.e., farm-to-fork, wood-based panels trusted value-chain, and securing autonomous driving function at the edge, and we also briefly introduce the expected benefits that the proposed FISHY architecture may bring. For the sake of clarity, Table 2 summarizes the expected benefits, along with the relevant stakeholders.

### 5.1. Farm-to-Fork Supply Chain (F2F)

During the last years, consumers’ demand for “safe” food, including organic, is skyrocketing; thus, producers, manufacturers, sellers, and end-users are often struggling to verify the accuracy of data across the whole supply chain of products (i.e., from the farm to the fork). Yet, consumers, especially within such niche markets, such as organic food, are increasingly willing to pay for products that provide this information. To date, solutions have revolved around EU certifications and regulations, both of which add costs, are hard to enforce, create everlasting bureaucratic processes, and, finally, can be confusing to consumers. The challenge here is to obviate the need for a central trusted intermediary and, instead, develop a decentralized process that, without the need of intermediaries, achieves the same or even better (in terms of accuracy, trust, evidence, complexity) functionality as today’s solutions. In this particular agricultural supply chain scenario, all the interested stakeholders will be able to receive information about the conditions under which the products have been cultivated, stored, and transported during their entire lifetime. This use case elicits requirements from real supply chain business processes in the agri-food sector that are involved in cultivating, tracking, tracing, and selling perishable goods.

For the sake of overall understanding, the lifecycle of agri-food products, from their production to consumption point, is quite complex and involves a large number of actors and services, and may generate a vast amount of data. For example, inside the farm, a perishable product could generate large volumes of related data (e.g., environmental conditions, utilization of fertilizers, date of plantation and harvest, water resources consumption). During transportation, data related to the preservation conditions (refrigerator temperature and humidity), shipment details, and truck route (GPS data) until final destination can be traced and stored in a distributed ledger, excluding the possibility of non-repudiation. Additionally, data can be created in other intermediary places, such as distribution centers, keeping data with respect to warehouse conditions, final destination, responsible employee, etc. Finally, all the data can be processed, and made available to consumers in the supermarkets.

### 5.2. Wood-Based Panels Trusted Value-Chain (WBP TRUST)

The manufacturing of wood-based panels is conducted as a continuous process, involving the feeding of raw materials (wood and resins from external suppliers), their processing (through heat and pressure), and either final panels delivery (sanding and cutting) or further processing (such as surfacing with decorative papers from external suppliers). The panels are then supplied to industrial clients, large or small, in the sectors of furniture manufacturing, flooring production, construction systems, or interior design applications (e.g., wall paneling).

The requirements from those B2B clients, in terms of product quality, standards compliance, and service levels, are more and more demanding. Moreover, the diversity, in terms of the product mix (different sizes, thicknesses, finishes, or other product characteristics), is also increasing. This context led involved companies to develop manufacturing strategies in novel production plants, aiming at creating a digitally connected and collaborative approach to manufacturing, exchanging data throughout the whole value-chain (upstream and downstream) in a fast, reliable, and secure way. This strategy means bridging the gap between two realities—information technology (IT) and operational technology (OT)—to ensure security, resilience, and availability at all levels. This is achieved through an integration architecture that considers different layers, from the shop floor level to a corporate level (holistic view of different production plants), also including an external layer that facilitates data sharing and automation from a value-chain perspective (from raw materials suppliers, logistics providers, and machinery maintenance companies to industrial clients).

Being a rather traditional industry, production plants typically rely on a wide number of different machineries from different suppliers, some of them old and with outdated software. These conditions challenge data from distinct contributors in the production line to be properly extracted and integrated, thus making data sharing not so easy. Moreover, a proper deployment of those novel manufacturing strategies requires strict connectivity guarantees of those machines through sensors and IoT devices, in order to enable data flows at the plant level (manufacturing floor), at the company level (between different plants), and in an ecosystem perspective (with suppliers and clients). This also poses significant challenges to ensuring the security, integrity, and reliability of these data flows, and the resilience of the whole set of systems in place. Assessing the risks and vulnerabilities of all the equipment (machinery, sensors, PLCs, and others), and ensuring the cybersecurity of all the connected devices, and, consequently, preventing attacks and incidents while guaranteeing the availability (uptime) of the production plants, is of paramount importance. Additionally, from a value-chain perspective, ensuring the security by design of the different integration layers (at the plant level, at a corporate level, and at an external level) is key to foster trust, and to stimulate data sharing/mining, as well as the development of novel value-added services. Unfortunately, the lack of a reference architecture and of a comprehensive framework for value-chain systems connectivity poses several relevant challenges to the implementation of such a vision. Indeed, nowadays, data at an OT level is not shared with external entities.

### 5.3. Securing Autonomous Driving Function at the Edge (SADE)

With the increasing number of electronic, intelligent embedded systems and connectivity in cars, plus the impending revolution of the fully connected and autonomous cars, security is becoming a major concern in the automobile industry, regulators, and also the public. Indeed, “*84-percent of automotive professionals have concerns that their organizational cybersecurity practices are not keeping pace with evolving technologies*” [83].

In practice, the aspects that have been widely adopted by manufacturers showing strong concerns about cybersecurity, fall around both software in the automotive supply chain and the level of connectivity in the connected car. On one hand, OEMs rely on hundreds of providers for many of the embedded systems. This requires controlling hundreds of different software versions within the car, posing challenges around SW verifications and attestation, and how to regularly maintain and patch this software and integrate it in a unified way within the automobile cybersecurity framework. On the other hand, automobile systems are now more exposed to remote risks and tampering.

In order to tackle these two major challenges, OEMs are introducing more-complex systems into the cars, such as firewalls and gateways, which are new systems that are also prone to risks, updates, and maintenance by the OEMs. Besides the best practices, such as security testing prior to release, patching or managing a homogenous/consistent secure software development lifecycle becomes more and more challenging. Usually, the industry relies on patching, dynamic security testing, and penetration testing during its development phase, to address some of them, but once the car is released, the increasing volume of electronics (sensors, actuators, gateways, firewalls, and ECUs) makes it difficult to maintain (i.e., development and patching) the systems. This also leads to concerns about new functions that OEMs may consider deploying, especially when sensitive user information is stored in any embedded system (this applies specially to any biometric information of the user). In this context, the automobile industry tends to shift most of the intelligence to the cloud, in order to simplify all car electronics. This trend is being boosted by upcoming technologies, such as 5G, C-V2X, 802.11P, and edge computing.

## 6. Conclusions

This paper puts the focus on a highly attractive scenario, that is, cyber resilience provisioning in supply chains built on complex ICT systems. The contribution starts by identifying the key challenges, still demanding specific attention by the scientific community, justified by a thorough review of the current literature in those key research areas that are strongly linked to the main objective. Then, from a technical view, the paper introduces a functional architecture aimed at addressing the specific challenges previously identified by that particular supply chain context.

Beyond the functional architecture definition, it is worth mentioning the use case scenarios shown as key examples, where the proposed architecture may be deployed at, as well as the benefits such a deployment may bring in to these scenarios and, what, indeed, is the main rationale for the architecture definition.

This paper may be read as both a wide survey of existing contributions in the well-identified research areas aligned to the main supply chain paper target, as well as a benchmarking report, in terms of either architectural design and/or real benefits for other similar initiatives.

## Figures and Tables

**Table 1:** Relevant research areas for IoT complex supply chains including current advances and key issues.

Research Area	State-of-the-Art	Key Issues
Information Security Assessment	Device security requirements, environment threat levels, assessment process characterization	Quality of security metrics, metrics properties, general model
Policy-based Systems	Traditional manual configuration or some tools for limited automatization	Full protected scenario, high- to low-level policies translation in non-simple scenarios, configuration orchestration
Trust Monitoring	Remote attestation procedure (TPM)	Considering virtualized environments, traffic attestation (at packet-node level)
Authentication and Authorization	Edge devices security provisioning is an open challenge	Different authentication levels considering constrained edge systems, distributed data sharing, secure nodes discovery, secure virtualization
Threat and Anomaly Detection	IDS is commonly deployed in IT infrastructures	No trained systems rather limited configurable systems, using ML for training
Threat Intelligence and Information Sharing	SIEM solutions	Current SIEM limitations to face IoT systems, using MISP
Identity Management and Accountability	Centralized solutions, recent DLT-based IdM solutions	No holistic view, exploit existing solutions to edge systems
Intent-based services	Current automatized management solutions based on policies or intents	Deploy intent-based solutions to orchestrate security actions in a human friendly scenario
Artificial Intelligence	Several network management solutions and NLP platforms exist, benefiting from AI	Adopting AI to facilitate overall system smartness and autonomy, considering intents orchestration and NLP, deciding where decisions should be taken

**Table 2:** Use cases benefits and key stakeholders.

Use Case	Benefits	Stakeholders
Farm-to-Fork	(1) designing a tailored solution for farm-to-fork-like supply chain scenarios; (2) enabling transactions between actors and devices belonging to different (isolated) IoT silos; (3) enabling trade of resources within the farm-to-fork supply chain in an automated, trusted, and decentralized way; (4) adding an audit service for involved business parties to identify and verify points of failure affecting product quality as products are transported from the farm to the selling point.	Producers, farms, manufacturers, sellers, logistics, and consumers
Wood-based Panels Trusted Value-Chain	(1) enabling security at each integration layer; (2) providing security, integrity and reliability of data flows and systems resilience; (3) ensuring the cybersecurity of all connected devices, preventing attacks and incidents and guaranteeing the availability of the production plants; (4) making the security by design of the different integration layers a must; (5) enabling regular bidirectional data flows with external entities by enforcing the necessary trust levels.	Raw materials suppliers, logistics providers, machinery maintenance companies, industrial clients, cloud providers.
Securing Autonomous Driving Function at the Edge	(1) adding new functionalities (facial key and sensors secure environment); (2) certification of the different sensors and actuators integrated in the car; (3) enabling a proper, secure and private data collection; (4) minimizing the security requirements impact on the car by offloading the security applications into the edge.	OEMs, software developers, edge providers, cloud providers, end users, security developers

## Abbreviations

The next abbreviations are used in this paper.

AI	Artificial Intelligence
AWS	Amazon Web Services
B2B	Business to business
COPS	Common Open Policy Service
DLT	Distributed Ledger Technology
ECA	Event-Condition-Action
EDC	Enforcement and Dynamic Configuration
F2F	Farm-to-Fork
IBN	Intent-Based Networking
ICT	Information and Communication Technologies
IdM	Current identity Management
IDS	Intrusion Detection Systems
IETF	Internet Engineering Task Force
IoT	Internet of Things
IPFS	InterPlanetary File System
IPS	Intrusion Prevention Systems
IPSec	Internet Protocol Security
IRO	Intent-Based Resilience Orchestrator and Dashboard
IT	Information Technology
JSON	JavaScript Object Notation
MAC	Media Access Control
MDGSM	Method for Designing Good Security Metrics
MISP	Malware Information Sharing Platform
ML	Machine Learning
NED	Network Edge Device
NETCONF	Network Configuration Protocol
NFVO	NFV Orchestrator
NFV	Network Function Virtualization
NIST	US National Institute for Standards and Technology
NLP	Natural Language Processing
NSF	Network Security Function
NSP	Network Service Provider
OEM	Original Equipment Manufacturer
ONOS	Open network Operating System
OT	Operation Technology
PBNM	Policy Based Network Management
PLC	Programmable Logic Controller
SADE	Securing Autonomous Driving Function at the Edge
SACM	Security Assurance and Certification Management
SCM	Security and Certification Manager
SDN	Software Defined Networking
SEN	Secure Edge Node
SIA	Secure Infrastructure Abstraction
SIEM	Security Information and Event Management
SNMP	Simple Network Management Protocol
SME	Small and Medium Enterprise
SUPA	Simplified Use of Policy Abstraction
TLS	Transport Layer Security
TM	Trust Manager
TPM	Trusted Platform Module
VNF	Virtualized Network Function
WBP TRUST	Wood-Based Panels Trusted Value-Chain
XML	Extensible Markup Language
YANG	Yet Another Next Generation
